# Numerical Investigation of Enhanced High-Intensity Laser–Matter Interactions in Nanowire-Coated Conical Targets

**DOI:** 10.3390/nano15231763

**Published:** 2025-11-24

**Authors:** Laura Ionel, Cristian Viespe

**Affiliations:** Laser Department, National Institute for Laser, Plasma and Radiation Physics, Atomistilor Str. 409, 077125 Magurele, Romania; laura.ionel@inflpr.ro

**Keywords:** nanostructured targets, nanowires, laser intensifier, spatio-temporal analogy, ultrashort laser pulses, ultraintense lasers

## Abstract

Nanostructured targets are increasingly used as key components in high-power laser–matter interaction experiments due to their ability to substantially enhance laser absorption, increase ion/electron generation, or boost the secondary radiation (THz, X-ray, etc.) in accordance with the actual scientific requirements in ultraintense regimes. Their tailored surface features influence the way the energy is deposited in the material, leading to significantly enhanced interaction effects compared to the flat conventional targets. In this study, we numerically investigate the mechanisms of laser field intensification occurring in the interaction between an ultraintense laser pulse and a nanostructured conical target. In order to provide a complex spatio-temporal description of the laser intensity evolution in the interaction area, we developed a 2D finite-difference time-domain model in accordance with the relative spatial extension of the pulse. The laser field intensification is numerically investigated in the vicinity of the laser matter interaction point considering four different materials of the nanopatterned conical targets and variable laser beam parameters in order to determine the optimum conditions to streamline the laser field enrichment in the laser solid targets interaction area. The numerical results show that the designed nanostructured profile of the internal cone target walls under imposed particular conditions induces a highly controllable increase in laser field intensity. Consequently, this enhanced field localization highlights the essential role of nanostructured design in advancing ultraintense laser applications that require efficient energy coupling and extreme field concentrations.

## 1. Introduction

The interaction of ultrashort and ultraintense pulsed lasers with structured matter provides a versatile tool for generating energetic particles, coherent radiation, and high-energy-density plasmas [1,2,3,4]. At relativistic intensities, the efficiency of energy transfer from the incident laser to the target critically determines the output and quality of secondary sources [5]. Conventional planar targets, however, present a limited absorption due to specular reflection, leading to incomplete coupling between the laser field and the dense plasma. In order to address this limitation, considerable attention has been devoted to engineering advanced target architectures capable of enhancing local field intensities, sustaining volumetric heating, and guiding hot-electron transport [6].

In the last decade, nanowire arrays have emerged as one of the most promising classes of structured targets [7,8,9,10]. Their high aspect ratio, sub-wavelength spacing, and sharp tips induce strong localized field enhancement via the “lightning-rod” effect, promoting near-complete absorption of incident laser energy. Previous studies have shown that nanowires sustain deep laser penetration and volumetric energy deposition [11], resulting in efficient hot-electron generation and enhanced ion acceleration [12]. Meanwhile, conical targets are remarkable for their geometric ability to concentrate laser energy toward a focal point, a concept that has been exploited in fast-ignition inertial confinement fusion, high-harmonic generation, and particle acceleration [13]. The combination of geometric focusing and extended interaction length within the conical cavity allows for the further intensification of the electromagnetic fields and confinement of energetic particles [14].

Integrating nanowire arrays with conical geometries presents a challenging hybrid approach, which may bring potential advantages in nanoscale field enhancement process and macroscopic geometric focusing systems. When laser pulses propagate inside nanowire-coated cones, the interplay between nanostructure-induced absorption and cone-guided confinement can lead to highly localized energy concentration in the tip area, thereby creating favorable conditions for secondary particle and radiation sources. Despite the strong potential of these architectures, a detailed understanding of the underlying electromagnetic field distribution within such hybrid targets requires particular investigations. The Finite-Difference Time-Domain (FDTD) method provides a powerful numerical tool capable of addressing this novel challenge. By directly solving Maxwell’s equations in the time domain, the FDTD method enables accurate modeling of the spatio-temporal evolution of laser fields in customized geometries and heterogeneous media. It is particularly designed to resolve sub-wavelength features, such as nanowires, and to solve the coupling between nanoscale field enhancement and macroscopic focusing effects. While previous numerical studies have explored either nanowire arrays or conical geometries separately, a comprehensive analysis of nanowire-based conical targets using the FDTD method has not yet been systematically reported.

In this work, we employ the FDTD method to investigate the propagation and spatio-temporal distribution of high-intensity laser fields inside nanowire-coated conical targets. We analyze the role of nanowire parameters (length, diameter, spacing) and cone geometry (height, length) in shaping the electromagnetic field at the interaction point and its contribution in laser enhancement process. Particular attention is given to the field localization in the vicinity of the cone tip, where energy concentration leads to subsequent plasma formation and particle acceleration. The results provide perspective insights into the mechanisms of enhanced field coupling in nanowire-based conical targets and offer design guidelines for optimizing structured targets in high-power laser–matter interaction experiments.

## 2. Methodology

### 2.1. Numerical Model and Setup

A two-dimensional FDTD numerical model was developed to investigate the spatio-temporal aspects of the laser beam intensification process in an ultraintense laser-microstructured target interaction system under particular conditions. This approach provides an explicit numerical solution to Maxwell’s curl equations and it is extensively applied in a wide range of integrated quantum optics applications due to its lack of theoretical constraints. In this framework, Maxwell’s equations describe the interdependence between the temporal variation in the electric field and the spatial evolution of the magnetic field. The simulations were carried out using the FULLWAVE package of the commercial software RSoft Photonics, Version 8.1.0.0.3 [15] which enables scalable modeling for diverse laser system schemes and extends the standard FDTD method through several advanced computational features. The present numerical investigation was dedicated to analyzing the spatio-temporal characteristics of electromagnetic field propagation in complex geometries relevant to nanomaterials. In subsequent studies, specific nonlinear phenomena—such as self-phase modulation, filamentation, and ionization—will be individually examined to explain their influence on the field–matter interactions at the nanoscale. In particular, the model can conveniently define nonlinear and dispersive materials within the software interface, allowing a comprehensive description of the field dynamics. As a result, the model can provide diverse material responses while efficiently reducing the overall simulation time. In the present study, the modeled spatial region was assumed to be free of conduction currents and isolated charges.

The interaction of the ultraintense laser pulse with the proposed nanostructured conical target was numerically investigated using the RSoft FullWAVE module. FullWAVE implements the FDTD method to solve Maxwell’s curl equations in the time domain:

(1) $$\nabla \times E=-\mu_{0}\frac{\partial H}{\partial t},\;\;\;\nabla \times H=J+\varepsilon_{0}\frac{\partial E}{\partial t}$$

where *E* and *H* are the electric and magnetic fields, *ε*_0_ and µ_0_ are the permittivity and permeability of free space and *J* represents the current density induced in the target. For nanostructured metallic or semiconductor targets, the dielectric response is usually modeled using Drude-Lorentz formalism which tackles both the plasmonic effects and laser field enhancement:

(2) $$\varepsilon \left(\omega \right)=\varepsilon_{\infty }-\frac{\omega_{p}^{2}}{\omega^{2}+i\omega \gamma }+\sum_{j}\frac{f_{j}\omega_{0j}^{2}}{\omega_{0j}^{2}-\omega^{2}-i\omega \Gamma_{j}}$$

with *ω_p_* and *γ* denoting the plasma frequency and collision rate (scaled by default to a common scale), *ε_∞_* the high-frequency permittivity and the Lorentz terms (*ω*_0*j*_*,* Γ*_j_, f_j_*) describing bound electron resonances. The incident laser pulse was induced as a time-dependent Gaussian beam, characterized by its spatial waist, central frequency, temporal duration and focal position. In FullWAVE, the auxiliary differential equation (ADE) method can be used to integrate dispersive materials:

(3) $$\frac{dJ}{dt}+\gamma J=\varepsilon_{0}\omega_{p}^{2}E$$

The total displacement current is expressed as:

(4) $$D=\varepsilon_{0}\varepsilon_{\infty }E+P_{dispersive}=\varepsilon_{0}\varepsilon_{\infty }E+\frac{J}{i\omega }$$

From spatio-temporal point of view, the FDTD algorithm solves Maxwell’s equations by discretizing them both in space and time using central difference operators. The equations are solved by using the Yee’s mesh [16], giving the possibility to compute the components of the electric and magnetic field at specific spatial mesh points. Moreover, the FDTD implementation adheres to the Courant–Friedrichs–Lewy (CFL) stability condition, ensuring that the temporal step allows sufficient propagation time for electromagnetic information within each spatial discretization unit, expressed as:

(5) $$\Delta t\leq \frac{1}{c}\frac{1}{\sqrt{\frac{1}{{\left(\Delta x\right)}^{2}}+\frac{1}{{\left(\Delta y\right)}^{2}}+\frac{1}{{\left(\Delta z\right)}^{2}}}}$$

where *c* denotes the speed of light and *Δx*, *Δy* and *Δz* represent the grid spacing along the three spatial dimensions.

To prevent non-physical reflections at the computational boundaries, Perfectly Matched Layer (PML) conditions [17] were implemented. In our study, the PML thickness was set to 0.4 µm on each spatial axis.

FullWAVE allows calculation of local field enhancement E_loc_/*E*_0_ at nanowire tips or cone surface and it calculates the energy absorption according to:

(6) $$P_{abs}=\frac{1}{2}R\{J\cdot E^{*}\}$$

As previously mentioned, the FullWAVE package of RSoft (Synopsys) is a full-vector solver based on Finite-Difference Time-Domain (FDTD) method which primarily works with the electric field (*E*) and magnetic field (*H*) values at each computational grid point. From these, it can compute the Poynting vector, which represents the power flow density:

(7) $$S=\frac{1}{2}Re(E\times H^{*})$$

where H* is the complex conjugate of *H*. Due to the fact that Maxwell’s equations are linear with respect to the electric and magnetic fields, the absolute amplitude of the laser source does not affect the spatial or temporal field distribution, but only scales the resulting field magnitudes proportionally. Consequently, FullWAVE does not assume a specific absolute source power. It normalizes the source amplitude to 1 V/m (or arbitrary units).

However, it is important to mention that the results can be rescaled analytically to any desired physical power by applying the quadratic scaling rule generally expressed as:

(8) $$P\propto {\left|A_{Source}\right|}^{2}$$

To obtain absolute values corresponding to a physical input power $P_{in}^{\left(abs\right)}$, the results must be rescaled as follows:

(9) $$E^{\left(abs\right)\;}\left(r\right)=\sqrt{\frac{P_{in}^{\left(abs\right)}}{P_{in}^{\left(rel\right)}}}E^{\left(rel\right)}(r)$$

(10) $$I^{\left(abs\right)}\left(r\right)=\frac{P_{in}^{\left(abs\right)}}{P_{in}^{\left(rel\right)}}I^{\left(rel\right)}(r)$$

where $P_{in}^{\left(rel\right)}$ is the power associated with the normalized simulation. In FullWAVE module, fields are normalized with *E*_0_ = 1. Absolute intensity follows:

(11) $$I=\frac{1}{2}c\varepsilon_{0}{\left|E\right|}^{2}$$

For a target intensity *I_phys_* = 10^18^ W/cm^2^, the scale is:

(12) $$E_{0}=\sqrt{2I_{phys}/(c\varepsilon_{0})}$$

The normalized vector potential at λ = 800 nm is $a_{0}\approx \sqrt{I\left[\frac{10^{18}W}{{cm}^{2}}\right]}\lambda [\mu m]$. Thus, our arbitrary units map directly to *a*_0_ once *I*_0_ is chosen. Intensities plotted as arbitrary units convert by multiplying with *I*_0_. Relative distribution and ratios are invariant by linearity.

In our work, this normalization approach offers several advantages. First, it simplifies the computational process by eliminating the need to specify an arbitrary or potentially uncertain source power prior to simulation, reducing the numerical processing resources and decreasing the simulation time offering a scalable model which is widely applicable in specific experiments. This approach allows users to focus on determining key optical characteristics such as reflection, transmission, coupling efficiency, or field confinement being independent of the absolute source amplitude. Secondly, normalized computations permit comparability and scalability, enabling direct comparison between different structures, material configurations or excitation conditions without the misaddressing influence of distinct power levels. Finally, because FullWAVE results can be readily rescaled to any physical intensity or power, normalization enhances the flexibility and generality of the simulation framework for both research and micro and nanostructures design applications. By expressing results in normalized units, the software facilitates efficient analysis of preconfigured structures while preserving the ability to convert outcomes to real-world power levels when required.

It is known that the type nanostructured target material determines how strongly the laser field is concentrated at the interaction point because it sets practically the wire’s optical properties. The material controls the nanowire’s linear optical response, ionization and thermo-mechanical response all these strongly influence the field enhancement during a high-intensity laser pulse nanostructured target interaction. Linear dielectric function, plasmonic response, collision frequency, ionization potential, thermal, mechanical and damage threshold play the essential role in describing the field enrichment model in certain conditions.

**Figure 1:**
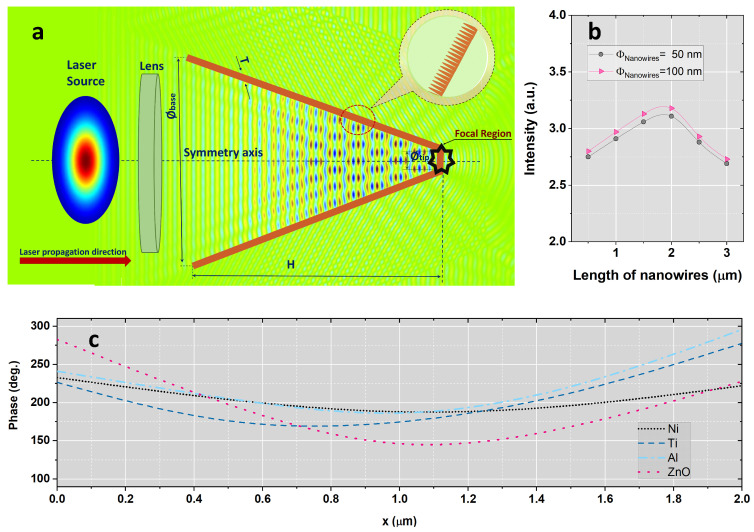
(**a**) Schematic of the FDTD simulation domain for laser interaction with a nanowire-coated conical target. (**b**) Intensity evolution in the presence of nanowires length variation in the range of 500 nm to 3 µm and (**c**) phase variation along the *x*-axis in case of Ni material at 25 fs pulse duration and f# = 2.5.

In this study, the laser field enhancement process was investigated by varying the pulse duration in the range of 5–55 fs in case of four different target materials. The schematic representation of the laser nanostructured conical target interaction is illustrated in Figure 1. As the standard length unit in RSoft is the micrometer, the proposed scalable design considered a Gaussian laser source with diameters D of 30 µm, 60 µm and 90 µm corresponding to f-numbers (f#) of 2.5, 1.2 and 0.8, respectively, and a normalized electric field amplitude of *E*_0_ = 1. This value corresponds to a relative input laser intensity of I_0_ = |*E*_0_|^2^ = 1.0 in the dimensionless units used by the FullWAVE package of RSoft. The source was linearly polarized with central wavelength of 800 nm and, for computational simplicity, a thin lens with focal length of 75 µm was used to focus the beam instead of an off-axis parabolic mirror, which is typically employed in high-intensity laser-matter interaction experiments. The laser source was modeled as a sinusoidal carrier wave modulated by a Gaussian envelope, mathematically expressed as:

(13) $$E\left(t\right)=exp\left[-A{\left(t-t_{d}\right)}^{2}\right]sin\left(\frac{2\pi c}{\lambda }t+\phi_{0}\right)$$

where *λ* is the central wavelength (800 nm), *τ* denotes the pulse duration which is dependent on the number of the wave cycles, *t_d_* is the temporal delay proportional to *τ*, *A* represents the chirp coefficient (in µm^−2^) and *ϕ*_0_ is the initial phase, set zero at the Gaussian peak.

A linearly polarized laser pulse is incident along the cone axis and propagates toward the apex. The inner surface of the cone is uniformly covered with vertically aligned nanowires of diameters ϕ_1_ = 50 nm and ϕ_2_ = 100 nm, length in the range of 500 nm to 3 µm, and spacing 10 nm. Perfectly matched layer boundary conditions are applied at the domain edges to absorb outgoing radiation. The simulation grid resolves both the nanowire dimensions and the cone surface, enabling calculation of the spatio-temporal evolution of the electromagnetic fields and the field localization near the cone tip.

### 2.2. Determination of Optimal Geometrical Parameters

In this approach, we considered the structured conical targets having a nanopatterned internal wall profile with four distinct materials: Nickel, Silicon, Aluminum and Zinc Oxide. The modeled target geometry, shown in Figure 1a, consisted of a conical structure with height (H) of 100 µm, a base diameter (ϕ_Base_) of 100 µm, a tip diameter (ϕ_Tip_) of 10 µm and a wall thickness of 5 µm. The computational domain was defined as 200 µm × 180 µm, with a background refractive index of 1, a grid spacing of 0.04 µm and a time step of 0.028 µm, consistent with Courant stability criterion. Considering the latest work related to the laser field enhancement and following the optimized parameters established in [18,19,20,21] the target internal wall profile was configured with nanowires at nanometric scale having the diameters ϕ_1_ = 50 nm and ϕ_2_ = 100 nm and the length in the range of 500 nm to 3 µm (Inset of Figure 1a). These specific geometrical configurations were selected as the most efficient from the perspective of laser intensification, in accordance with the prior studies on the spatial distribution of focused laser fields [18,19].

Besides the electromagnetic pulse intensity evolution analysis within the focal region after propagation through the nanostructured conical target, this approach also includes a numerical study of the spatio-temporal (S-T) field distribution in the focal region in order to determine the temporal instants at which the peak field intensity occurs. These numerical computations facilitate the establishment of an analytical correlation between the laser field intensity evolution and the degree of S-T coupling within the focal region. The numerical results demonstrate that spatio-temporal discrepancy defined as a deviation between the spatial and temporal extents plays a critical role in the intensity enhancement process of ultrashort pulses. Accordingly, this numerical approach contributes to a deeper understanding of the S-T equivalence of few-cycle laser pulses by proposing a robust computational methodology for analyzing intensity evolution in the focal region of nanowire-coated conical targets.

## 3. Results and Discussions

### 3.1. Spatial Distribution and Laser Intensity Evolution

**Figure 2:**
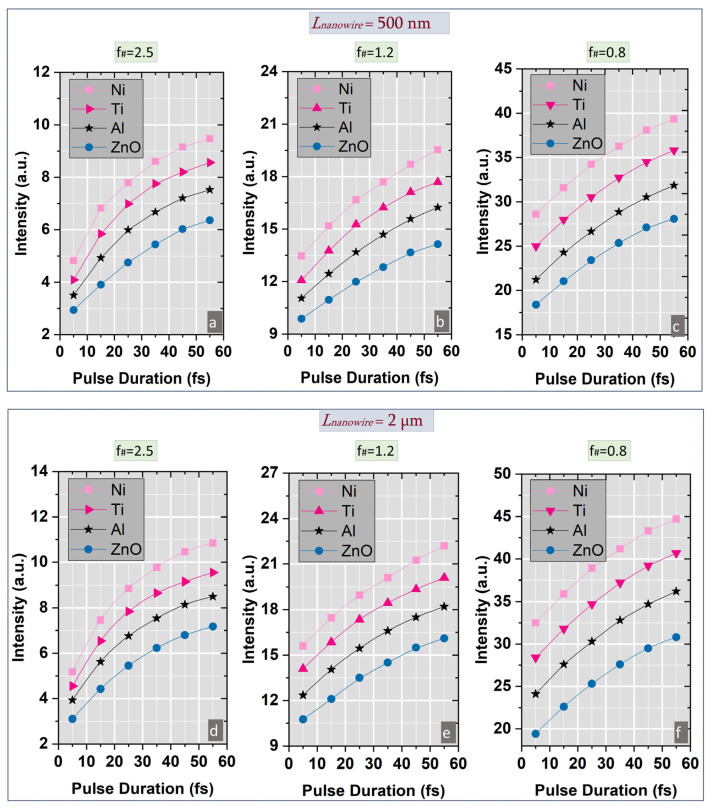

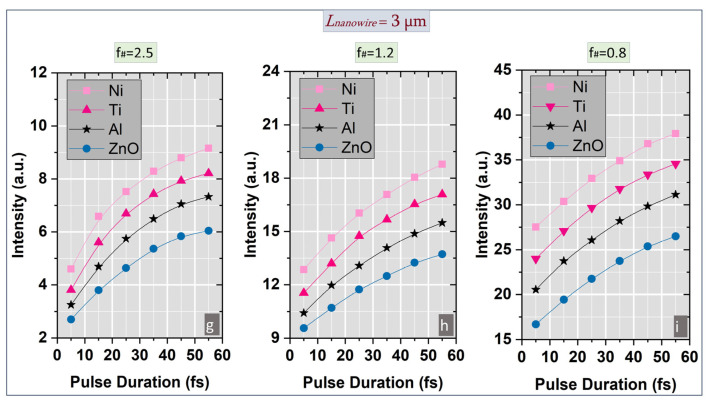
The evolution of the laser field intensity in the focal point after the propagation of a Gaussian laser beam through a nanostructured conical target in case of pulse duration variation for different f-numbers: (**a**,**d**,**g**) f#_1_ = 2.5; (**b**,**e**,**h**) f#_2_ = 1.2; (**c**,**f**,**i**) f#_3_ = 0.8.

A detailed numerical investigation of the spatial distribution of the electromagnetic field was carried out in the vicinity of the laser-nanostructured conical target interaction point, in the presence of pulse duration variation in the range previously mentioned with the aim of identifying the specific temporal instants corresponding to the peak intensity values (Figure 2a–i). Initially, a comprehensive analysis of the electromagnetic field intensity behavior was conducted for a focused laser beam propagating through a nanopatterned conical target for all four materials included in the study within a specific region around the focal point and three different numerical apertures (f#_1_ = 2.5, f#_2_ = 1.2 and f# = 0.8). Prior to the analysis of laser pulse intensity evolution, a preliminary study was conducted to determine the optimal material characteristics and structures dimensions in order to identify the configurations yielding the highest field enhancement in the focal region. Based on the laser nanowire-coated conical target interaction system illustrated in Figure 1a, the influence of the target material and size on the intensity evolution of the laser field at the interaction point was evaluated under pulse duration variation down to few-cycle regime. The temporal monitors, uniformly distributed through the interaction zone, provided data corresponding to the highest laser field values, which were subsequently analyzed. As shown in Figure 1b, the maximum intensity value was obtained for nanowires with 2 µm length and 100 nm diameter in case of nickel. This initial investigation was therefore essential for establishing the optimum nanowires dimensions employed in subsequent simulations. In addition to the intensity analysis, the numerical model could provide essential data concerning the phase evolution of the longitudinal electric field factor by applying a discrete Fourier transform (DFT) frequency algorithm. As illustrated in Figure 1c, the smallest phase variation along the x axis was obtained for nickel, in accordance with the intensity results previously assumed.

**Figure 3:**
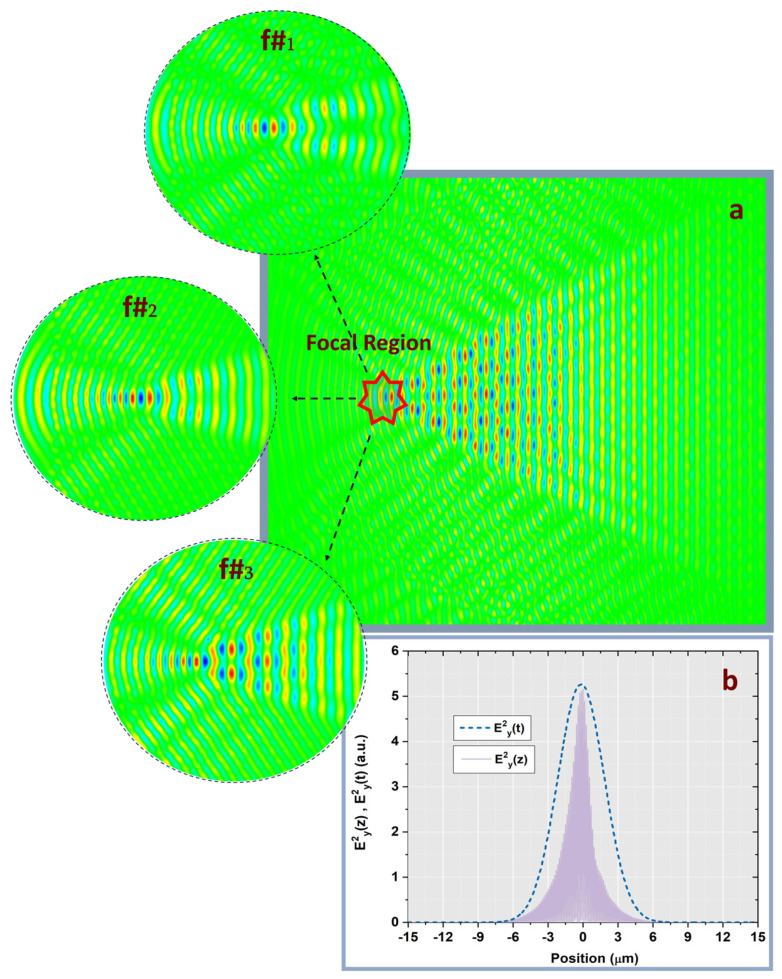
(**a**) Spatial distribution of the laser field after propagation through a nanowire-based conical targets for three different f-number values (f#_1_ = 2.5, f#_2_ = 1.2 and f#_3_ = 0.8). (**b**) Relation between the temporal behavior of the square of the electric field envelope and the spatial extent of the laser pulse along the propagation axis for nickel material at 25 fs pulse duration and an f-number of 2.5.

A detailed investigation of the laser field intensity dynamics in the vicinity of the focal point was performed as a function of pulse duration for each f# value and for the optimized nanowires dimensions previously determined. Compared to the case of nanostructured conical targets, additional studies reveal that the microstructured targets produced intensity values approximately twice as high as those of the unstructured surfaces. Among the current materials analysis, Ni exhibited the highest intensity enhancement for all three numerical apertures and for all pulse durations values (Figure 2a–i). By extending the numerical analysis to the extreme values of the nanowire’s length range (500 nm and 3 µm, respectively), we observe a general decrease in intensity values of approximately 12% for *L_nanowire_* = 500 nm and 15% for *L_nanowire_* = 3 µm for all f# values compared to the optimum case of *L_nanowire_* = 2 µm. For particular case of *L_nanowire_* = 2 µm, it was observed that Ni produced approximately two times greater intensity values than those obtained for ZnO over the pulse duration range. Overall, a similar trend in intensity evolution with the pulse duration was observed for all four target materials under equivalent input conditions. For a constant emitted energy, the laser field intensity in the focal spot was higher for the 90 µm beam than for the 30 µm and 60 µm beams, owing to the influence of the numerical aperture on the focusing of the peripheral and paraxial rays after lens propagation (Figure 3a). For all three f# values, the laser intensity increased monotonically with the chromatic dispersion (dn/dλ) of the target material, underscoring the role of the dispersion in the field enhancement process. Consequently, nickel, possessing the highest chromatic dispersion value, yields the most intense field value.

The numerical results show that when an ultra-short and ultraintense laser interacts with a nanostructured target (e.g., nanowires coated substrate), the geometry of the nanowires plays a key role in determining how strongly the electromagnetic field is enhanced. Specific nanowire dimensions (length, diameter, spacing) lead to higher intensity enrichment because they concentrate the incident laser energy into localized fields or plasma excitations. If the nanowire diameter and length correspond to the resonance conditions for localized surface plasmon resonances (LSPRs), the incident laser energy couples actively to these modes. At resonance, the laser energy becomes confined in the vicinity of the wire tips or gaps, resulting in intense fields which can be an order of magnitude higher than the incident field. Moreover, these resonances depend on the wire’s architecture and dimensions and the aspect ratio. Long and thin nanowires may act like optical antennas. The sharper the tip or the smaller the radius of curvature, the stronger the electric field concentration at the tip. This is a geometric effect which is independent of the material resonance but with high impact on the laser intensity enhancement process. Also, an array of nanowires spaced close together can lead to constructive interference and stronger local fields. The optimum spacing depends on the laser beam parameter such as wavelength and polarization. Some geometries minimize reflectivity and behave as graded index structures allowing more energy to enter the target. At high intensities, the enhanced fields can further lead to nonlinear electron emission, field ionization and plasma formation, aspects which are envisaged in our future work. Once the plasma is generated, it can focus the laser energy through the enhanced field, depending on the wire architecture and plasma parameters.

Overall, additional study demonstrates that the use of a nanowire coated conical target can lead to intensity enhancement of up to threefold compared with no-structured surfaces for all three numerical apertures considered. The results clearly indicate that in laser-matter interaction process intensity enhancement is strongly dependent on the geometrical configuration of the interaction region and the optical properties of the implied materials.

### 3.2. Temporal Field Dynamics

Considering the prior investigation of the S-T characterization of ultrashort pulse evolution in the focal area [21], the present study examines the S-T analogy of the laser field for f#_1_ = 2.5, f#_2_ = 1.2 and f#_3_ = 0.8 under pulse duration variation extending down to few-cycle regime. The temporal evolution of the ultrashort laser pulses for different pulse durations (τ) was analyzed in the vicinity of the focal spot, using data provided by the temporal monitors which are positioned along the propagation axis by introducing a spatio-temporal correlation analysis. The temporal full width at half maximum (FWHM) values were calculated for each case under similar input conditions. In order to determine the temporal envelope of the electromagnetic field, Ey^2^(t), the temporal coordinate was converted into a spatial one by using the Minkowski relation (*s* = *c*×*τ*, where *c* represents the speed of light, *τ* denotes the pulse duration and *s* is the spatial extension of the laser pulse), having the laser field spatial extension expressed along the propagation direction (Figure 3b). The numerical computations were performed in normalized units of *c × τ* (with 1 µm ≈ 3.33 fs). The temporal simulations employed the same conditions imposed in the preceding intensity dynamics analysis.

The results reveal that the temporal FWHM variations correspond directly to the spectral broadening of the laser pulse. The numerical results further presented reveal that the highest ratio between spatial and temporal FWHM occurred for Ni, for all three f# cases under pulse duration variation.

### 3.3. Spatio-Temporal Analogy

A complementary investigation of the space-time (S-T) analogy was conducted for the considered laser source maintaining the same conditions previously assumed—specifically, the same structural target materials, nanostructured profile and f-number values consistent with the geometrical configuration of the laser-matter interaction scheme. Analyzing the evolution of the laser field in the focal region, for f# equal to 2.5 a slight increase in the electromagnetic FWHM pulse was observed for all investigated cases. These findings indicate that the input beam profile plays an essential role in defining the spatio-temporal dynamics of ultrashort laser pulses in few-cycle regime.

To further quantify the relation between the spatial and temporal extent of the focused ultrashort pulses, the relative spatial extension (RSE) parameter, as defined in [22], was accordingly employed. The RSE was calculated using the expression:

(14) $$\frac{u}{n}=s_{FWHM}c\tau_{FWHM}$$

where *n* represents the refractive index of the target material, *s_FWHM_* represents the spatial FWHM of the focused pulse envelope, the terms *τ_FWHM_* corresponds to the temporal pulse duration at the focal point and c is the speed of light.

**Figure 4:**
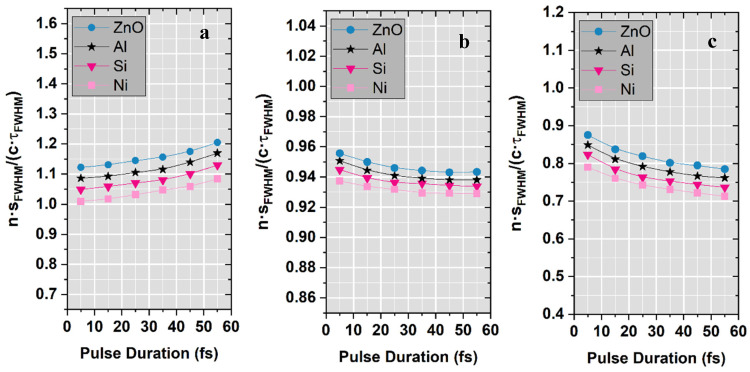
Variation in the relative spatial extension of the focused laser beam as a function of pulse duration in focal point after propagation through nanowire-coated conical targets for all four materials investigated and different f-numbers: (**a**) f#_1_ = 2.5; (**b**) f#_2_ = 1.2; (**c**) f#_3_ = 0.8.

The study related to the RSE approach is comprised in Figure 4. The results show that RSE presents a similar trend for all four target materials exhibiting slightly higher values at f# = 2.5 and lower values at f# = 0.8. Notably, for f# = 2.5 the RSE parameter increases with pulse duration for all considered materials, whereas for f# = 1.2 and f# = 0.8 it decreases significantly. This opposite behavior emphasizes the interplay between the focusing conditions and material dispersion in shaping the spatio-temporal characteristics of the pulse.

Overall, this study confirms that for higher f-numbers both intensity and RSE increase with pulse duration while for lower f-numbers, intensity exhibits a similar growth but RSE presents a noticeable decrease.

### 3.4. Benchmarking with Published Experiments (Scope and Comparability)

To associate our numerical predictions with experimental observations, we benchmark the relative enhancement between structured and unstructured surfaces—under identical optical and focusing conditions—against reported data for nanowire arrays and cone-like targets irradiated by femtosecond pulses. Various experiments have demonstrated that nanowire arrays exhibit enhanced absorption and secondary emission at relativistic or near-relativistic intensities, consistent with local field amplification at tips and inter-wire gaps [8,9,10,11]. Our simulations reproduce the corresponding qualitative trends:

(i) Increasing f/# leads to smoother focusing and a larger effective Rayleigh range, producing a monotonic rise in on-axis intensity and relative spatial extension (RSE);
(ii) Decreasing f/# results in stronger geometrical confinement and phase curvature, which enhances peak intensity but reduces RSE, indicative of tighter spatio-temporal coupling;
(iii) Material-dependent dispersion, represented by ε(ω), modulates the enhancement in a manner consistent with experimental findings on metallic and semiconductor nanowires.

Given that experimental facilities employ varying pulse energies and contrast levels, we emphasize relative enhancement ratios rather than absolute intensity values. For comparable geometries and focusing conditions, our simulated structured/flat intensity ratios fall within the factor-of-few enhancements commonly observed for nanowire and conical targets in the pre-plasma regime. We note, however, that subsequent plasma dynamics following ionization may further influence the measured yields [8,9,11]. Accordingly, we pose the present study as a predictive framework for the electromagnetic field build-up preceding plasma onset, thereby providing initial conditions relevant to subsequent plasma-mediated absorption and secondary emission processes.

In summary, the numerical analysis demonstrates that the spatial extent of the focused ultrashort laser pulse is governed by the Rayleigh range and remains shorter than its temporal duration for all investigated scenarios. Furthermore, under specific focusing and material conditions, higher pulse intensities can be achieved. Future investigations are envisaged to study material-specific morphological effects and local laser field modulation mechanisms that may contribute to intensity enrichment in the interaction area.

## 4. Conclusions

An FDTD numerical model was developed to explore the correlation between spatio-temporal dynamics and the intensity evolution of focused laser beams in an ultrashort pulse laser—nanostructured conical target interaction scheme, with pulse durations extending into the few-cycle regime. The results indicate that the input beam profile, numerical aperture, and target material properties are key parameters influencing laser field enhancement at the focal point. Within the S–T framework and consistent with the Rayleigh range extension, the simulations confirm that shorter and more intense pulses can be obtained under well-defined conditions. Overall, this numerical approach provides valuable insights into the mechanisms of laser field intensification, offering guidance for the design and optimization of ultrashort, ultraintense laser–nanostructured target interactions in the few-cycle regime.

Outlook: Further numerical analyses of plasma dynamics in ultraintense laser fields—emphasizing the morphological characteristics, material-specific parameters, and underlying mechanisms responsible for the spatial modulation of the electromagnetic field that amplifies the laser pulse intensity within the interaction domain—are planned to be conducted in future work.

## Data Availability

The original contributions presented in this study are included in the article. Further inquiries can be directed to the corresponding author.
